# Intensive Outreach for Mental Health: Young People’s Experiences of an Intensive Outreach Model on Recovery and Engagement

**DOI:** 10.1007/s10597-024-01387-z

**Published:** 2025-01-06

**Authors:** India Bellairs-Walsh, Maria Nichterlein, Ben Assan, Robyn Stargatt

**Affiliations:** 1 School of Psychology and Public Health, La Trobe University, Plenty Road & Kingsbury Drive, Bundoora, VIC 3086 Australia; 2 Present Address: Orygen, 35 Poplar Road, Parkville, VIC 3052 Australia; 3 Present Address: Centre for Youth Mental Health, The University of Melbourne, Parkville, VIC 3010 Australia; 4 Present Address: School of Health Sciences, Swinburne University of Technology, Hawthorn, VIC 3122 Australia; 5 Child and Youth Mental Health Service, Austin Health, Marian Drummond Annex, PO Box 5555, Heidelberg, VIC 3084 Australia

**Keywords:** Adolescent, Community mental health services, Assertive community treatment, Qualitative

## Abstract

Engagement with traditional mental health services can be particularly challenging for young people experiencing severe and complex mental health problems. Assertive community treatment-based services providing mobile outreach, such as Intensive Mobile Youth Outreach Services (IMYOS), operate across Australia to support these young people’s mental health needs in the transition to adulthood. Past research on IMYOS has focused on quantitative outcome measures, and young people’s experiences of this type of model are poorly understood. This study explored youth service users’ experiences of an IMYOS program, focusing on the model and intervention aspects perceived as barriers and facilitators to their overall recovery and service engagement. Semi-structured, in-depth interviewing was undertaken with nine young people aged 16–19 years (*M* = 17.61 years) who were current or recent service users of an IMYOS program in Melbourne, Australia. Data were analysed using thematic analysis. Having a therapeutic space, clinicians’ specialised expertise, use of an outreach and community-oriented approach, and care continuity and availability across settings were program aspects identified by young people as facilitating their recovery and service engagement. Program aspects perceived as barriers to recovery and engagement were clinical unsuitability and ineffectiveness of the program and interventions, and conflicts between personal autonomy and assertive care. Young people’s perceptions of clinical suitability and therapeutic relevance influenced their evaluations of the overall effectiveness of the IMYOS service, which subsequently impacted their engagement. The findings provide opportunities for IMYOS clinicians to enhance young people’s recovery and engagement outcomes and have implications for the improvement of this innovative service for at-risk young people.

**Supplementary Information:**

The online version contains supplementary material available at 10.1007/s10597-024-01387-z.

## Introduction

Approximately 3–5% of Australian children and adolescents experience severe and complex mental health problems that are associated with high co-morbidity and psychological distress (Fowler et al., 2010; Lawrence et al., 2015). If left unaddressed, these can be devastating to long-term developmental pathways; resulting in prolonged educational and employment instability, psychosocial disability, and functional impairment across the lifespan (Lawrence et al., 2015; McGorry et al., 2012). While early intervention can help to minimise these impacts, numerous factors can make service engagement significantly challenging for young people with complex and chronic mental health needs. These include interactions between developmental changes, psychopathology, and reduced functional capacity, ‘help-negation’ effects involving decreased help-seeking despite increased psychological distress, and systemic and structural barriers to service access (Hall et al., 2001; Radez et al., 2021; Wilson, 2010). Such engagement challenges can lead to reduced treatment efficacy and poorer psychosocial outcomes (Kim et al., 2012), leaving young people with complex mental health problems without appropriate support.

Intensive Mobile Youth Outreach Services (IMYOS) aim to address some of these challenges. IMYOS programs are a specialist component of tertiary-level public mental health services across Victoria, Australia, positioned within Child and Youth Mental Health Services (CYMHS). Similar models operate throughout Australia and are delivered through state government funding (e.g., Assertive Mobile Youth Outreach Services in Queensland). These services provide intensive mental health outreach to young people with significant mental health needs, who have typically experienced difficulties engaging in less intensive services and may benefit from more flexible treatment in their own environment. Young people in this cohort are often described in the literature as “hard-to-reach” or “difficult-to-engage”, in recognition of the challenges in establishing service engagement and a therapeutic alliance (Vijverberg et al., 2017). Many young people receiving care from IMYOS have a high prevalence of psychiatric co-morbidities, have frequent in-patient admissions, have experienced significant traumas, present with challenging, at-risk, or suicidal behaviours, and may lack sufficient social support; making them highly vulnerable in the transition to adulthood (Chia et al., 2013; Purcell et al., 2011). IMYOS thus focuses on providing mental health care to young people who stand to lose the most from service disengagement, with the aim of addressing disengagement risks, optimising functioning, and returning the young person to their developmental pathway (Chia et al., 2013; Cosgrave et al., 2008; Fowler et al., 2010).

As a wrap-around, community-based mental health program, IMYOS is based on the spirit of Assertive Community Treatment (ACT) (Bond & Drake, 2015; Bond et al., 1995, 2001; Firn, 2007). The theoretical underpinnings, service delivery model, and core interventions of IMYOS have been described extensively elsewhere (Assan et al., 2008; Chia et al., 2013; Ryall et al., 2008; Schley et al., 2011). However, in brief, IMYOS operates from a range of developmental, trauma, attachment, neurobiological, psychodynamic, family, and systems theories, with interventions delivered at the system and individual level through assertive outreach. Emphasis is on a ‘safety-first’ approach, assertion opposed to coercion, the therapeutic alliance, flexibility and responsiveness, and a holistic treatment model over diagnostic categorisation. Multifaceted, individualised interventions are based on client needs, and include case management, risk and crisis management, individual/group psychotherapy and pharmacotherapy, family counselling/therapy, alcohol and substance counselling, intensive secondary consultation (e.g., with carers and/or schools), and practical support and community capacity building (e.g., assisting school return or engagement with appropriate social networks). In line with a developmental approach, IMYOS supports the involvement of young people’s families and carers, and can deliver mental health care to a young person in the absence of the young person’s agreement where clinically appropriate, with informed consent from their legal authority. Like ACT-based services, shared caseloads across clinicians and continuous and rapid patient coverage, including after-hours and weekend on-call consultation, are notable features. IMYOS teams are multi-disciplinary and generally include a mix of psychologists, social workers, occupational therapists, psychiatric nurses, and a consultant psychiatrist. Caseloads are reported to be between 6–10 (Assan et al., 2008) and 8–9 clients (Schley et al., 2011) per clinician, depending on demands. The average length of IMYOS involvement for youth users has been reported to be 9 months (*SD* = 6.7) at one site (Assan et al., 2008) and 12.6 months (*SD* = 6.8) at another (Schley et al., 2008).

Past research on ACT services for young people has identified significant improvements in cognitive, emotional, behavioural, and somatic outcomes, as well as in functional domains including interpersonal, school-based, employment, and residential placement (Baier et al., 2013; Camilleri et al., 2017; Godley et al., 2002; McGrew & Danner, 2009; Ogden & Hagen, 2006; Vijverberg et al., 2017). Retrospective and prospective quantitative studies have assessed the utility of IMYOS-type programs in Victoria and Queensland, and identified positive outcomes such as increased full-time education attendance and global functioning scores, reduced psychiatric admissions and time in hospital, and decreased risk of harm to self and others including suicidal ideation, violence, and crime (Chia et al., 2013; Daubney et al., 2021; Schley et al., 2008). Retention and engagement rates of young people in IMYOS are also markedly high – reported to be up to 100% at one IMYOS site (Assan et al., 2008). In 2019, The Royal Commission into Victoria’s Mental Health System recommended that this early intervention service be expanded, outlining the potential for financial savings from reduced psychiatric hospitalisations, youth suicides, and chronic mental health-related disability (State of Victoria, 2021).

Despite the reported beneficial therapeutic and engagement outcomes of IMYOS programs, there is little evidence on what specifically contributes to these outcomes. One quantitative study conducted at an IMYOS site identified that factors associated with the therapeutic alliance and engagement, such as ‘collaboration’, ‘perceived treatment usefulness’, and ‘client-therapist interactions’ were associated with better outcomes for young people at discharge (Schley et al., 2012). It has also been speculated that features of the IMYOS model that are consistent with ACT services, including assertive outreach, and holistic, client-centred approaches that emphasise a strong therapeutic relationship and clinician responsiveness, may play a role (Assan et al., 2008; Firn, 2007; Fowler et al., 2010). However, no studies on intensive assertive outreach approaches for young people have examined how these models may assist outcomes from the position of IMYOS users themselves. Exploring young people’s perspectives are essential to ensure that services sufficiently address their needs (McGorry et al., 2014). Furthermore, improving our understandings of the IMYOS components that may underpin clinical utility and recovery in this cohort can better equip clinicians and service providers in responding to the diverse engagement needs of this group, address barriers to the therapeutic relationship, and contribute to the progression of this innovative service.

## Methods

### Study Design

This qualitative study utilised individual, in-depth interviews to explore youth service users’ experiences of an IMYOS program. Specifically, their perspectives on the IMYOS model and intervention aspects perceived as barriers and/or facilitators to their overall recovery and engagement with this service. The theoretical framework and orientation informing data collection, analysis, and interpretation was aligned with an experiential, mixed inductive-deductive approach (Braun & Clarke, 2012); focusing on representing participants’ expressed realities, while also drawing from literature on the therapeutic alliance and engagement.

### Setting, Recruitment, and Sample

Participants were young people receiving treatment from an IMYOS program servicing the North-eastern metropolitan Melbourne catchment area, located in Victoria, Australia. Current and recently discharged IMYOS service users were invited to take part. Inclusion criteria were being aged 16 years or above, being able to read, understand, and speak English, and a minimum service engagement of four months of the program. Exclusion criteria were experiencing an acute stage of illness (e.g., hospitalisation, active psychosis/mania, suicidal crisis).

Sampling employed both purposive and convenience features, with the IMYOS team producing a pool of potential participants who met the recruitment criteria. Current service users were provided with the study information by their IMYOS clinician during their usual clinical contact, and discharged users were contacted by the IMYOS team who sought permission for the first author to approach them for participation. Potential participants were then contacted directly by telephone/SMS to establish rapport and explain further information about the study. The study information was emailed or posted to those who expressed interest, and interview arrangements were made with those who consented to take part. Although the IMYOS clinicians initially informed clients of the study, they were blind to their decision to participate, allowing the expression of young people’s opinions on the service delivery in a confidential setting. Recruitment ceased when the data were sufficiently rich and novel to address the study aim, support the analysis, and generate new understandings, as per the guidelines of information power (Malterud et al., 2016).

Nine participants between 16–19 years took part (*M* = 17.61 years, *SD* = 1.09), with five identifying as female, three as male, and one as transgender male. Five participants were currently receiving treatment from IMYOS while four had been discharged, with the mean time since discharge being 8.42 months (*SD* = 5.95). Overall, the mean length of involvement with the program was 1.74 years (*SD* = 1.16).

### Materials

A semi-structured interview schedule was used to elicit perspectives on the program. The schedule was developed in consultation with the IMYOS team, who identified areas of clinical interest that would provide valuable information about the service delivery model. The interview schedule is provided in Supplementary File 1.

### Procedures

Interviews were conducted by the first author across metropolitan Melbourne in a one-on-one, face-to-face setting. Participants nominated preferred interview times and locations; seven occurred in participants’ homes, one in a suburban public library, and another in a university psychology clinic. This in-field approach served to enhance participant comfort and rapport and reflect the principles of outreach services. All interviews were recorded and undertaken in one session, with lengths ranging from 75.35 to 203.60 min (*M* = 142.71 min). The schedule was followed until no new information was elicited and the properties of the responses had been thoroughly explored, however, flexibility was given to explore unforeseen responses. Empathic communication strategies were used throughout the interviews, including attentive, respectful listening, and reflecting the content and emotion of participants’ responses (Alston & Bowles, 2012). The first author transcribed the audio recordings and offered participants the opportunity to review their transcript to check for accuracy, provide clarity, and give their approval for inclusion. This process aimed to promote a cooperative stance and ensure that participants’ voices were represented to their satisfaction (Mero-Jaffe, 2011). All participants provided written informed consent, approved their full transcripts for inclusion, and were paid AUD $30 remuneration for their time.

### Data Analysis

The qualitative software package NVivo 11 (QSR International Pty Ltd, 2017) was used to assist with coding and data management. Data were analysed following the processes of thematic analysis described by Braun and Clarke (2006, 2012, 2013). First, the primary author reviewed the transcripts, field notes, and audio recordings multiple times to promote immersion in the data. They then conducted line-by-line coding to generate initial codes, and coded for as many potential themes and patterns as possible. Next, they collapsed and clustered the codes into overarching themes, paying attention to frequent and significant codes, and the underlying relationships between codes, subthemes, and themes. Thematic tables and maps were constructed, and memo-writing was undertaken to assist with theme development and refinement. While use of the Consolidated Criteria for Reporting Qualitative Research (COREQ) checklist (Tong et al., 2007) has known limitations (Braun & Clarke, 2024), we have included this in Supplementary File 2 as a space to provide elaboration on the methods and analysis. Additionally, a reflexivity statement outlining the positionality of the first author who conducted the interviews and analysis is provided in Supplementary File 3.

## Results

Four themes relating to the IMYOS model and intervention components represented facilitators of young people’s recovery and engagement, including *“Having a therapeutic space”*, *“Clinicians’ specialised expertise”*, *“Using an outreach and real-world approach”*, and *“Continuity and availability of care across settings”*. Two themes – *“Clinical unsuitability and ineffectiveness”* and *“Conflicts between personal autonomy and assertive care” –* reflected barriers to young people’s recovery and engagement.

### IMYOS-Related Facilitators to Recovery and Engagement

#### Having a Therapeutic Space: *“I can let out all my thoughts”.*

Nearly all participants expressed that communicating about their symptoms and problems was helpful and necessary for their recovery. Compared to formal therapeutic spaces, the non-traditional support environment of IMYOS was viewed by young people as promoting open communication and disclosure: *“It’s [a] pretty kind of relaxed atmosphere… you can talk about just anything… it became easier for me to just talk freely”* (P7). Participants saw the relationship with their clinician as one that gave them a valued space to connect and debrief. In this space, they could release and process emotional experiences: “Sometimes I’ll be like ‘Oh, this happened, it was sh*t’ and other times I’ll be like ‘Yeah, I’m having a really good week’” (P2). Participants described experiencing a sense of emotional unburdening in this therapeutic space – *“I can let out all my thoughts”* (P6) – which frequently offered reprieve and respite from re-living ongoing experiences of distress and trauma: *“[Talking about things] gets it out for the day or the week… that’s why I see [clinician] at the start of the week, so then it lasts me the week… so I don’t have to think about them things [trauma]”* (P1).

#### Clinicians’ Specialised Expertise: *“They know how to help”.*

IMYOS clinicians were viewed by participants as being able to help where others couldn’t, such as non-professional supports like parents or family members. Participants felt their clinicians had specific skills and knowledge to provide relevant and helpful advice that supported them with their specific challenges: *“I think the people [at IMYOS], they know how to help”* (P3); *“[They’ve] helped me cope with [the disorder and symptoms]… it’s been a lot better since I started seeing [them]”* (P2). Many participants described how their clinicians offered specific symptom management strategies, which they endorsed as positive: *“[They] would tell me ways to deal with [my anxiety]”* (P6); *“Talking about strategies and how you can make it better is always helpful”* (P4).

Participants often described experiencing difficulties with disclosing and discussing their mental health challenges. They described their clinicians as having the skills to be able to identify, sensitively broach, and encourage openness and communication about these challenges. One participant described how this made them feel more empowered and confident to discuss their mental health: *“They encouraged me to kind of talk about stuff, and they kind of take the lead from there, and they made it easier for me to talk about things and raise concerns”* (P7).

As well as helping to improve participants’ own mental health, the expertise of participants’ IMYOS clinicians often extended to benefitting their family members well-being and overall family relationships: *“[Clinician] knew what to do about my anxiety and how to cope with it, and how to sometimes deal with my Mum as well… so [they] kind of helped me not only deal with my problems, but try and also make it a little bit better for my Mum”* (P4).

#### Using an Outreach and Real-World Approach: *“They help get you out there”.*

The outreach approach of the IMYOS program was seen by participants as fundamentally beneficial to their situation and needs. Receiving accessible support at home addressed participants’ described engagement barriers, and they felt more inclined to maintain treatment: *“I think it’s been convenient… it’s put less pressure on me to go out when I was really anxious”* (P8); *“It was helpful in making sure that I [was] still having some kind of communication with someone… it can be really easy to just avoid [traditional services] and not go”* (P7). Participants again described how meeting their clinicians in everyday environments felt more organic and “relaxed” than traditional mental health settings, while maintaining a similar therapeutic purpose: *“[IMYOS] can just drive wherever, they can go places, which is also like more natural… it’s not like a ‘formal’ psychiatrist thing… it works similarly… but it’s just more laid-back”* (P8). These interactions in everyday settings were seen to minimise feelings of being “unwell” associated with traditional healthcare environments: *“When you go to like a doctor’s or something you feel sick… but then when I just see [clinician] it’s like just catching up with a friend”* (P2).

The outreach approach also assisted participants with functional ‘real-world’ activities. This was noted as a particularly beneficial element of the program that traditional support settings lacked: *“They help get you out there… I haven’t had anyone else try and help get me out there… I mean they have tried to help, but they haven’t actually gotten me out there”* (P9). Having the opportunity to apply therapeutic strategies in real-world situations, with the accompaniment and emotional support from clinicians, was viewed positively: *“[They] helped me communicate better… [clinician] would bring me to [restaurant] and help me order myself”* (P6). This community-oriented focus was seen to build confidence and skills that flowed to other domains, and improved participants’ symptoms and social inclusion: *“They’ve gotten me out of the house, and they’ve been making me more confident with the support they’ve given me… they would help me so I wasn’t alone doing it… I’m going out places now… I would have never gotten to school… I could barely make it through recess, and now I’m doing all my classes, and all my homework”* (P8).

The involvement of participants’ IMYOS clinicians across different domains of their life also promoted the recognition of ‘recovery barriers’ that participants felt they couldn’t identify themselves (e.g., ‘blind spots’), that may have been masked in traditional settings: *“It was more involved with all aspects of my life… having them kind of understanding what I did… things that you wouldn’t really think were a problem… I guess they noticed [it] in a lot of things”* (P7). These real-world insights into participants’ daily lives were viewed as providing opportunities to modify unhelpful behaviours and improve functioning: *“It’s good to have someone that can tell me things that I can’t see for myself… at the moment I try and get up around 9:00am and then go to bed around 10.30 pm… like not sleeping in super late”* (P2).

#### Continuity and Availability of Care Across Settings: *“It’s like ongoing help”*

Participants described how their IMYOS clinicians had ongoing communication with the wider IMYOS team, their families, and their schools. For participants, this promoted a sense of being scaffolded across a range of settings, and meant that if problems arose, they had a wider range of resources available to access: *“[Clinician] is there so Mum can always call if she thinks that I’m having problems and I can’t talk about it or something… [they] were [also] able to talk to the school counsellor and just say, ‘Keep an eye out’… so if I have any troubles and I’m not seeing [clinician] I can just go and talk to the mentor”* (P2).

IMYOS was also viewed as providing continuous support at times of need and crisis, with increased availability, responsiveness, and flexibility from clinicians a major advantage over traditional services: *“I can call them if I’m struggling, or text them. I couldn’t really do that with anyone else… they can make more appointments… they can try to see me more quickly”* (P3). When participants were navigating in-patient admissions, the *“ongoing help”* (P4) from clinicians assisted with difficult transitions in and out of hospital: *“I didn’t feel alone when I was in hospital because [clinician] was kind of managing all my paperwork… [they] came in to visit quite a few times as well. It was a lot better than when I was in there by myself”* (P9). Continuity of care between IMYOS team members was also valuable, and contrasted with traditional support environments where the loss of a professional resource due to absence was a risk: *“If [they’re] away there’s other people that I can either see or call… everyone sort of knows all the clients… normally you’d just have one person… and if they’re away… there’s not really anything you can do”* (P2).

This continuity of care across settings also meant that participants didn’t have to continuously “do the work” of re-explaining their situation, as different supports were kept informed and involved throughout: *“People will know how I’m feeling… I won’t have to explain it to [them] the next time I see [them]”* (P8).

### IMYOS-Related Barriers to Recovery and Engagement

#### Clinical Unsuitability and Ineffectiveness: *“It wasn’t really relevant”*

At times, strategies advised by participants’ IMYOS clinicians to assist their mental health were perceived as ineffective. This often arose from what participants viewed as a lack of focus towards targeting their specific challenges and needs. This perceived lack of focus or relevance resulted in reduced openness, communication, and problem disclosure: *“[They] do have some strategies but it’s more general than specific to what’s going on… [they don’t] really ask questions about what’s going on, it’s more just general conversation… it’s kind of harder to bring things up”* (P3). One participant described their experience with a different service, which they felt was able to provide more relevant support by asking more comprehensive, targeted, and in-depth questions: *“[Other support] asked me stuff that finds out how I think, and the way that I think, and how that affects the way things happen in my life right now… I just felt with [IMYOS] it was just kind of random. It wasn’t really relevant”* (P5).

Participants also outlined situations where the IMYOS interventions’ specific clinical goals, or rationale and purpose, were unclear to them: *“It never felt like [they were] trying to explain to me what [they were] trying to achieve”* (P5). This further exacerbated perceptions of unsuitability and ineffectiveness, both towards specific therapeutic tasks that clinicians suggested, and more broadly towards the program overall. For one participant, having a lack of clarity around their therapeutic goals in high-anxiety situations (e.g., leaving the house) was viewed as worsening their progress and recovery: *“I left the house with [clinician] a few times but… it just went downhill… it felt we didn’t really have that much of a plan when we went out”* (P9).

#### Conflicts Between Personal Autonomy and Assertive Care: *“I didn’t really have a choice”*

Some participants described experiencing a lack of autonomy in their decisions to engage with the IMYOS program. Overlapping with the previous theme, often those participants who perceived a lack of relevant or targeted support from IMYOS were the same who described feelings of pressure, frustration, and resistance towards engaging with the program. For example, Participant 5, who described feeling uninformed, also described feeling pressured to comply with this perceived unnecessary and unwanted help: *“I found it kind of annoying because I didn’t really have a choice that [they] came…”* (P5).

Similarly, some saw their IMYOS clinicians as possessing knowledge about their personal preferences and capabilities that went somewhat ignored. This resulted in feeling pressured to undertake activities that they felt mentally unprepared for, or that they anticipated they would be unable to cope with: *“Recently they’ve been pushing me… they want me to work up to things that I think are too soon… they want me to do things that maybe I’m not [as] interested [in] as they are*” (P8).

Conversely, other participants felt the assertive component of IMYOS had benefits, even if they didn’t recognise this at first. Some described experiencing initial resistance to the program in the early stages, but how over time, this changed to autonomous engagement due the program’s perceived effectiveness: *“I had no choice [earlier]… but now I’m choosing to do it… I think it’s been helpful*” (P6). Despite feeling pressure to engage in unwanted therapeutic tasks, some also believed these were, on the whole, beneficial to their long-term recovery: *“A few times I wanted to just stay home and they made me kind of go out, but I reckon it was for the best”* (P9). One participant detailed how the assertive nature of IMYOS was important in addressing their help-seeking challenges; ensuring they maintained connection to therapeutic support they may have otherwise disengaged from: *“I’m not good at making the initiative to seek help and so having that plan put in place was a positive thing”* (P7).

## Discussion

Young people identified several features of the IMYOS program that contributed to both their engagement with the service and overall recovery, including having an informal therapeutic space to process emotions, having access to specialised, skilled clinicians, improved treatment accessibility via outreach delivery, support with real-world engagement, and being scaffolded across settings with enhanced care continuity and availability.

Young people identified that the non-traditional outreach format is a much-preferred approach that not only increases treatment accessibility, but also enhances comfort, rapport, and open communication between service users and their clinicians. The client–clinician relationship was seen as a valued space for young people to process emotions, share problems and experiences, and receive management strategies – all of which helped to alleviate symptoms and distress. This reflects past research that identified the centrality of dialogue (i.e., the opportunity to talk and be listened to) as key to improving engagement in ACT-based services in adult populations (Priebe et al., 2005; Wright et al., 2011).

Importantly, receiving practical, ‘real-world’ and community-oriented support was noted to be a significant facilitator of symptom improvement and recovery. The opportunities to apply therapeutic tasks directly in the community seemed to promote functional gains, providing evidence for what has been previously speculated in the literature (Dilk & Bond, 1996). These resulting functional improvements described by participants could serve to mitigate the risk of experiencing long-term psychosocial disability and exclusion further along the lifespan.

Additionally, enhanced continuity and availability of care from IMYOS helped to provide wrap-around, integrated support for young people across a variety of settings. Given that cross-agency fragmentation in public health systems has been criticised (Orešković, 2016), such wrap-around support is an additional advantageous feature of IMYOS (and CYMHS services more broadly). Together, these findings reflect previous quantitative and qualitative literature on ACT services in adult populations that showed that continuous patient coverage and advocacy are beneficial features, and that the provision and reception of practical and social support, genuine conversation, and time commitment of staff promote service engagement (Bond et al., 1990, 1995, 2001; Chinman et al., 1999; Priebe et al., 2005; Watts & Priebe, 2002; Wright et al., 2011).

Despite the positive IMYOS features, perceived therapeutic unsuitability or irrelevance, including an absence of targeted support or clear therapeutic goals and rationales, were associated with negative evaluations of IMYOS effectiveness and beliefs around reduced clinical need. This created barriers to service engagement and overall symptom recovery, including tensions between young people’s personal autonomy and the assertive nature of the IMYOS program. Further contributing to these tensions were symptom experiences. For example, participants who lacked clarity on the purpose or focus of the intervention/s, or who experienced significant anxiety symptoms, tended to see limited value in the therapeutic tasks and describe resistance toward engaging. On the other hand, engagement trajectories were improved when young people saw value in the program, felt it was targeted appropriately, and could identify longer-term benefits. Thus, perceptions of clinical suitability and relevance, and subsequent evaluations of effectiveness/ineffectiveness, influenced young people’s engagement decisions.

The findings have important implications considering that an effective therapeutic alliance depends on the collaborative endorsement of therapeutic goals and tasks, and appreciation of their relevance and value (Bickman et al., 2004; Bordin, 1979). Factors associated with the therapeutic alliance, including beliefs about treatment usefulness, therapeutic collaboration, and having a “partnership model” are significant contributors to engagement and treatment outcomes in both adult and adolescent populations receiving mental health interventions (Karver et al., 2006; Meaden et al., 2004; Schley et al., 2012; Staudt, 2007; Watts & Priebe, 2002; Williams & Steer, 2011). It is also known that ACT-type teams often face conflicts between clients’ expressed desires and their long-term interests (Bond et al., 2001), and adult users of ACT services have previously expressed concerns with clinician paternalism (Chinman et al., 1999). Because assertive care involves the provision of treatment to patients who are vulnerable due to clinical severity and complexity, there may be instances where care provision is unwanted. Conversely, participants in this study also outlined how the assertive format of IMYOS can improve access to care for those experiencing significant barriers in attending traditional mental health services.

### Clinical Implications and Recommendations

Several key features of the IMYOS model were perceived by young people as advantageous over traditional mental health support and as contributing to enhanced psychosocial recovery. These features should be noted as core beneficial elements of such programs and should be attended to in their service development and delivery.

IMYOS operates with the therapeutic alliance as a central tenet; part of this objective is to ensure that therapeutic tasks are relevant and suitable to individual young people (and their families). However, difficulties in the therapeutic relationship can readily arise due to inherent challenges in the provision of care to those who may experience significant mental health, trauma, and interpersonal complexities. Thus, constant work on strengthening the therapeutic alliance must occur. Clinicians can focus on areas of individual concern, and develop clarity of, and agreement on, goals and tasks with the young people they work with – ensuring that as far as possible, these are collaboratively valued and endorsed. This could occur through fostering open and ongoing dialogue with frequent reassessment of suitability (e.g., ‘checking-in’), even if the proposed interventions are intended for presumably obvious beneficial reasons (e.g., school return).

At the operational level, incorporating systematic client feedback methods would be valuable, such as the *Partners for Change Outcome Management System* (Duncan, 2012; Duncan et al., 2017). Such practices are integrated into the ongoing psychotherapeutic process, and involve client-led and defined outcome targets, routine client feedback, and collaborative ongoing sharing and discussion of progress and alliance instruments (Duncan, 2012; Duncan et al., 2017). Utilising a *shared decision-making protocol* or *decision support tool* intervention may have benefits for populations experiencing severe mental health problems (Thomas et al., 2021), by helping to promote service user autonomy and the delivery of individualised, person-centred, value- and evidence-based care. There have been promising early results on the feasibility and utility of shared decision-making protocols with young people, including those with complex mental health needs (Langer et al., 2022; Simmons et al., 2021). However, more evidence is needed to demonstrate positive impacts on patient outcomes, including treatment engagement.

### Strengths and Limitations

It is possible that the youth IMYOS users who participated in this study had better service experiences and/or recovery outcomes, compared to those who chose not to take part, which may be reflected in participants’ narratives. We were also conscious that current IMYOS users may have been reluctant to disclose if they had experienced any difficulties or problems with the service, perhaps due to fears of being perceived as “complaining”. Social desirability effects, such as participants responding in accordance with their perceptions of what the researcher “wanted to hear” were, of course, also possible. Strategies were undertaken to mitigate these possibilities, including adopting a non-hierarchical interviewing approach to facilitate good rapport, balancing the interview questions (e.g., asking about both perceived barriers and facilitators), emphasising IMYOS clinician blindness and confidentiality, and including discharged service users in the sample. However, we recognise and embrace the inherent participant and researcher subjectivity in this work, including the co-construction of realities.

The study had several strengths. The interviewer was conscious of participants’ barriers to participation, so in-field interviews that replicated the IMYOS delivery format were conducted to help maximise participant comfort and accessibility. Further, to the authors’ knowledge, this is the first qualitative study conducted on young people’s experiences in the Australian IMYOS context. This cohort of young people typically face exclusion from research settings, thus, it was important to centre their voices and experiences of the service, to ensure IMYOS sufficiently addresses their needs.

### Future Directions

Future research in the IMYOS context may wish to quantitatively examine the barriers and benefits of IMYOS programs with larger samples of young people. This could help to identify subgroups of service users for which these barriers and benefits are most applicable (e.g., particular diagnostic presentations), and advance the delivery of individualised, person-centred care. Examining the impact of ‘real-world’ therapeutic tasks in IMYOS on psychosocial functioning and recovery outcomes may also be beneficial and contribute to understandings of the therapeutic ‘mechanisms of action’ of IMYOS. Additional research with families of young people receiving IMYOS treatment, along with IMYOS clinicians, may also provide a deeper understanding of the factors associated with the program’s effectiveness and/or engagement.

## Conclusion

This study explored the experiences of young people with severe and complex mental health needs as they navigated treatment in a specialised ACT-based program in the Australian CYMHS system; specifically, their perspectives on the IMYOS model aspects that were viewed as facilitators or barriers to their recovery outcomes and service engagement – a previously unaddressed topic in the literature. Features of the IMYOS model, including accessible community-based support and continuous wrap-around care were seen as advantageous by young people and contributing to enhanced recovery and engagement, along with clinician skills and expertise in providing a safe therapeutic space that encouraged open communication and client empowerment. In contrast, perceptions of clinical unsuitability and ineffectiveness negatively impacted engagement and recovery, and were driven by a lack of therapeutic specificity, clarity, and young people’s symptom experiences. The findings provide valuable insight into young people’s experiences of the IMYOS model that can be used to inform improvements in intensive and assertive outreach-based programs, and improve engagement and recovery outcomes for youth service users.

## Supplementary Information

Below is the link to the electronic supplementary material.

Supplementary file1 (DOCX 19 KB)

Supplementary file2 (DOCX 32 KB)

Supplementary file3 (DOCX 19 KB)

## Data Availability

Due to privacy and ethical restrictions, interview transcripts are not able to be shared.
