# Supplementary material for: Intensive Outreach for Mental Health: Young People’s Experiences of an Intensive Outreach Model on Recovery and Engagement
Source: Community Ment Health J. 2025 Jan 6;61(4):693–703. doi: 10.1007/s10597-024-01387-z (PMC11968474; doi:10.1007/s10597-024-01387-z)
Supplement: Supplementary file 1 — Supplementary file1 (DOCX 19 KB) [file 10597_2024_1387_MOESM1_ESM.docx]

**Supplementary File 1**

Interview Schedule

**Introductory Statement**

Before we get started, I’d like to reiterate the purpose of the interview, which is to talk about your experience of working with the team from the [IMYOS] program and your perception of its usefulness, as well as what brought you there and how you’re going now. We hope that in hearing each young person’s unique experience we can gain a greater understanding of the outcomes of the program, how young people experience it, and their perceptions of supports and services.

Some people find it difficult to talk about what’s happened whilst others find easy, and for some this might be the first time they’ve spoken to someone outside the healthcare setting about certain things that have happened and how they understand them. Some of the questions may bring up thoughts or feelings that hadn’t occurred to you before. Some people also find that it can be helpful to talk about their experience even when it feels upsetting at first. However, it’s up to you how much you’d like to tell me, so as we talk just let me know how you’re going and if you wish to stop at any time. If there are any questions you don’t wish to answer just let me know and I’ll move on. The interview can go for quite a while, so if you’d like to take a break at any point, we can do that as well.
Are there any comments or questions you have about the process before we begin?

First, I’d like to ask a few questions about yourself, and to get some background information on what brought you to [IMYOS] and the events leading up to it.

**Introduction/Easing in**

1. What was your first contact with the team? Were there other things you were having difficulty with?
2. Were there other problems you were experiencing? How are things now? What problems are you experiencing now?
3. What is the problem you are having most difficulty with just now?

**Perceptions of support/IMYOS service**

1. What kind of help suits you best? What kind of help helps? What kind of treatment do you think you should receive? What are the most important results you hope to receive from the treatment?
2. Have you had any help in the past? Did you agree with the help that was advised? What hasn’t helped you? What has helped you?
3. Does your family, or others, agree with you about what help you should receive?
4. What difference will/does effective help make in your life?
5. Does anyone else around you need to receive help for the problem to go away?
6. Can you tell me about your work with [clinician] from [IMYOS]? What does he/she help you with? Where do you meet? Is there anything different about the help you get from [clinician] compared to other professional help you’ve received?
7. What happens when you meet with [clinician]?
8. Do you find your work with [clinician] from [IMYOS] helpful? If yes, how is it helpful?
9. Are there any negative things about seeing [clinician] from the [IMYOS] team?
10. Before you started seeing [clinician], did you receive support from another service? Or was there some overlap between those helping you? If yes, did you notice a difference in the kinds of help provided?
11. Are there any differences between your previous workers and [clinician] from the [IMYOS] team?
