# Supplementary material for: Intensive Outreach for Mental Health: Young People’s Experiences of an Intensive Outreach Model on Recovery and Engagement
Source: Community Ment Health J. 2025 Jan 6;61(4):693–703. doi: 10.1007/s10597-024-01387-z (PMC11968474; doi:10.1007/s10597-024-01387-z)
Supplement: Supplementary file 2 — Supplementary file2 (DOCX 32 KB) [file 10597_2024_1387_MOESM2_ESM.docx]

**Supplementary File 2**COREQ Checklist

| **Topic** | **Item No.** | **Guide Questions/Description** | **Reported on Page No.** |
| --- | --- | --- | --- |
| Domain 1: Research team and reflexivity |  |  |  |
| *Personal characteristics* |  |  |  |
| Interviewer/facilitator | 1 | **Which author/s conducted the interview or focus group?**   The first author. | 3 |
| Credentials | 2 | **What were the researcher’s credentials? E.g., PhD, MD.**   Bachelor of Psychological Science – BPsySc. | Supp. File 3 |
| Occupation | 3 | **What was their occupation at the time of the study?**  Full-time student, Bachelor of Psychological Science Honours program; part-time Academic Support Worker. | Supp. File 3 |
| Gender | 4 | **Was the researcher male or female?**   Female. | Supp. File 3 |
| Experience and training | 5 | **What experience or training did the researcher have?**   The first author held a Bachelor of Psychological Science qualification at the time of the study, with research ethics and methods training conducted as part of their degree. This was the first study they conducted independently as part of their Honours program. They had some interviewing experience from a previous role involving conducting telephone interviews for data collection with participants across Australia. | Supp. File 3 |
| *Relationship with participants* |  |  |  |
| Relationship established | 6 | **Was a relationship established prior to study commencement?**  The first author was unknown to participants prior to the commencement of the study and recruitment. | 3 |
| Participant knowledge of the interviewer | 7 | **What did the participants know about the researcher? E.g., personal goals, reasons for doing the research.**   The interviewer introduced themselves during initial contact with participants, explaining their role as an Honours student and the purpose and aims of the study. This was reiterated at the beginning of each interview. | N/A |
| Interviewer characteristics | 8 | **What characteristics were reported about the interviewer/facilitator? E.g., bias, assumptions, reasons and interests in the research topic.**   See Supplementary File 3 for the researcher’s positionality. | Supp. File 3 |
| **Domain 2: Study design** |  |  |  |
| *Theoretical framework* |  |  |  |
| Methodological orientation and theory | 9 | **What methodological orientation was stated to underpin the study? E.g., grounded theory, discourse analysis, ethnography, phenomenology, content analysis.**  The theoretical framework and orientation informing data collection, analysis, and interpretation was an essentialist/realist, experiential, mixed inductive-deductive approach. Data was analysed following the approaches of thematic analysis outlined by Braun and Clarke (2006; 2012; 2013). | 3, 4 |
| *Participant selection* |  |  |  |
| Sampling | 10 | **How were participants selected? E.g., purposive, convenience, consecutive, snowball sampling.**   Sampling was both purposive and convenience. The IMYOS team produced a pool of current and recently discharged clients of the program who met criteria for the study, and current clients were provided with the study information by their clinician, and discharged clients were contacted by the team who sought permission for the first author to approach them for participation. Those who met criteria and consented took part. | 3 |
| Method of approach | 11 | **How were participants approached? E.g., face-to-face, telephone, mail, email.**   Potential participants from the pool who met criteria and consented to be contacted by the first author about the study were contacted by telephone and SMS to establish rapport, explain further information about the study and determine interest. The first author then emailed or posted the study information to those who expressed interest, and where young people consented to take part, made arrangements with them for a suitable time and location for the interview. | 3 |
| Sample size | 12 | **How many participants were in the study?**   Nine. | 3 |
| Non-participation | 13 | **How many people refused to participate or dropped out? Reasons?**   The initial sample pool was 34 participants (10 current clients of IMYOS and 24 discharged clients) who were provided the initial study information by clinicians or who the clinicians attempted to contact about the study. 15 were excluded due to being uncontactable (*n* = 13), disinterest (*n* = 1), and psychiatric vulnerability (*n* = 1). The first author contacted the remaining 19 potential participants, 10 of which were excluded due to receiving no response or discontinuing communication with the researcher (*n* = 8), and declining consent (*n* = 2). | N/A |
| *Setting* |  |  |  |
| Setting of data collection | 14 | **Where was the data collected? E.g., home, clinic, workplace.**  Interviews were conducted metropolitan Melbourne by the first author in a one-on-one, face-to-face setting. Participants nominated times and locations; seven occurred in participants’ homes, one in a suburban public library, and another in the La Trobe University Psychology Clinic. This naturalistic, in-field approach reflects the fundamental principles of outreach services and served to enhance participant comfort and rapport. Furthermore, it allowed access to respondents that would have otherwise been out-of-reach in traditional research settings. | 3 |
| Presence of nonparticipants | 15 | **Was anyone else present besides the participants and researchers?**   The interviews were conducted in a quiet and private place, and no one else was present beside the participant and researcher in the interviews themselves. However, where interviews were conducted in young people’s homes, sometimes their families were also present in the home. | N/A |
| Description of sample | 16 | **What are the important characteristics of the sample? E.g., demographic data, date.**   Participants were aged between 16-19 years (*M*_age_ = 17.61 years, *SD* = 1.09), with five identifying as female, three as male, and one as transgender male. Five participants were current clients of the IMYOS program, while four had been discharged, with the mean time since discharge being 8.42 months (*SD* = 5.95). Overall, the mean length of involvement with the program was 20.87 months (*SD* = 13.94) or 1.74 years (*SD* = 1.16).  Further information about the sample includes:  Self-reported mental health diagnoses included mood and anxiety disorders (e.g., depression, bipolar disorder, generalised anxiety disorder, social anxiety disorder, agoraphobia, post-traumatic stress disorder), borderline personality disorder, and autism spectrum disorder. Sleep and eating disturbances, psychotic symptoms, and substance use, as well as neurological conditions were also disclosed. Psychiatric co-morbidity was a common feature, with almost all participants reporting between two and four concurrent conditions (*n* = 7). Nearly all were currently taking mental health-related medication (*n* = 8) and had experienced two or more psychiatric in-patient admissions (*n* = 7). | 3 |
| *Data collection* |  |  |  |
| Interview guide | 17 | **Were questions, prompts, guides provided by the authors? Was it pilot tested?**   The semi-structured, in-depth interview schedule is outlined in Supplementary File 1. The schedule was developed in consultation with the IMYOS team, who identified areas of clinical interest that would provide valuable information in relation to the service delivery model and to elicit perspectives on the program. The main topic areas relevant to this paper included perceptions of support and treatment, and experiences and engagement with the IMYOS program. The schedule was not piloted before the study; however, flexibility was given to match participants’ responses, encourage smooth transitions between lines of questioning, and allow exploration of unforeseen responses and themes. | 3 |
| Repeat interviews | 18 | **Were repeat interviews carried out? If yes, how many?**   No repeat interviews were conducted. | N/A |
| Audio/visual recording | 19 | **Did the research use audio or visual recording to collect the data?**   Yes, all interviews were audio-recorded with consent from participants. | 3 |
| Field notes | 20 | **Were field notes made during and/or after the interview or focus group?**   Yes, notes were taken during and after the interviews to identify key topics and ideas. Thematic tables, maps, and memos (both paper and digital) were also created during the data analysis process to help with theme generation, refinement, and consolidation. | 4 |
| Duration | 21 | **What was the duration of the interviews or focus group?**   Interviews ranged from 75.35 to 203.60 minutes (*M* = 142.71 minutes). | 3 |
| Data saturation | 22 | **Was data saturation discussed?**   We followed the guidelines of information power recommended by Malterud et al. (2016) – that is, that the data were sufficiently rich and novel to address the aim of the study, to support the analysis, and to generate new understandings. | 3 |
| Transcripts returned | 23 | **Were transcripts returned to participants for comment and/or correction?**  Yes, participants were offered the opportunity to review their transcript to check for accuracy, provide clarity, and give their approval for inclusion. This process promoted a cooperative stance and encouraged non-exploitation, by ensuring participants’ voices were represented accurately and to their satisfaction. | 3 |
| **Domain 3: Analysis and findings** |  |  |  |
| *Data analysis* |  |  |  |
| Number of data coders | 24 | **How many data coders coded the data?**   One (the first author), who conducted data/transcript coding, theme generation and development, and thematic refinement. | 4 |
| Description of the coding tree | 25 | **Did authors provide a description of the coding tree?**   Not applicable. | N/A |
| Derivation of themes | 26 | **Were themes identified in advance or derived from the data?**  Mostly derived from the data with a focus on representing participants’ expressed realities (i.e., inductive approach).  However, existing concepts in the literature (e.g., therapeutic alliance, engagement) helped to interpret the findings. | 3 |
| Software | 27 | **What software, if applicable, was used to manage the data?**  NVivo 11 (QSR International Pty Ltd., 2017) was used to store and manage the qualitative data. | 4 |
| Participant checking | 28 | **Did participants provide feedback on the findings?**   No. However participants were offered the option to receive a summary of the results at the conclusion of the study. | N/A |
| *Reporting* |  |  |  |
| Quotations presented | 29 | **Were participant quotations presented to illustrate the themes/findings? Was each quotation identified? E.g., participant number.**   Yes, all quotations were identified by the interview/participant number. All findings were illustrated with relevant thematic excerpts. | 4-6 |
| Data and findings consistent | 30 | **Was there consistency between the data presented and the findings?**   Yes, all data was interpreted within the Results and Discussion sections, and in relation to existing literature, novel findings, and clinical/service implications. | 4-8 |
| Clarity of major themes | 31 | **Were major themes clearly presented in the findings?**   Yes, there were six main themes that pertained to the central query of this paper – young people’s perspectives on the IMYOS model. Specifically, four themes related to program features that facilitated engagement and recovery, and two themes related to program features that were barriers to engagement and recovery. | 4-6 |
| Clarity of minor themes | 32 | **Is there a description of diverse cases or discussion of minor themes?**   Disconfirming case analysis was conducted throughout the analysis process to identify and account for data that was discrepant to the themes and patterns identified. Overall, discrepancies were reported as part of the properties of each relevant main theme. For example, within the final theme *“Conflicts between personal autonomy and assertive care”*, there were nuances in participants’ perceptions on the assertive nature of the program, which were explored within this overall theme. | 4-6 |

Developed from: Tong A, Sainsbury P, Craig J. Consolidated criteria for reporting qualitative research (COREQ): a 32-item checklist for interviews and focus groups. *International Journal for Quality in Health Care*. 2007. Volume 19, Number 6: pp. 349 – 357.
