# Supplementary material for: Intensive Outreach for Mental Health: Young People’s Experiences of an Intensive Outreach Model on Recovery and Engagement
Source: Community Ment Health J. 2025 Jan 6;61(4):693–703. doi: 10.1007/s10597-024-01387-z (PMC11968474; doi:10.1007/s10597-024-01387-z)
Supplement: Supplementary file 3 — Supplementary file3 (DOCX 19 KB) [file 10597_2024_1387_MOESM3_ESM.docx]

**Supplementary File 3**Reflexivity Statement

The primary author recruited participants, conducted interviews, and analysed and interpreted the data. They are an educated, white, middle-class, cis-gendered Australian woman. At the time of the study, they held a Bachelor of Psychological Science, and had experience in telephone interviewing in population-based research, community mental health volunteering, and within equity and diversity roles in the tertiary education sector. The study was conducted as part of the fulfilment of their Psychological Science Honours program and was the first qualitative study they had undertaken.

The primary author brought values of social justice and equity into the research, and a commitment to ‘providing a voice’ to the experiences of marginalised young people, who may have been excluded from research previously. The primary author holds beliefs about the period of adolescence as being one of ‘formative years’, and subsequently, beliefs in the importance of early intervention and person-centred, youth-friendly, and effective care; particularly that treatment experiences during this period can have significant impacts on long-term outcomes. The author also is cognisant that young people’s needs may not always be supported adequately, either by family, social, or institutional structures, including within the mental healthcare system, and takes these motivations into their health services research work, with the overall goal to enhance service users’ experiences and outcomes.

However, the primary author was an ‘outsider’ with limited experience – at the time, they were not a clinician and had an outsider’s knowledge of the public mental health system and the IMYOS program. While in some ways this was beneficial, in others, it was challenging. Due to their background and training in psychological science, the author was also aware of the possibility of mental health symptoms (e.g., varying insight levels; anxiety towards ‘exposure’ therapies) colouring treatment experiences for participants, and their narratives. To manage these tensions, the author took an approach of attempting to stay as ‘true’ as possible to the participants’ expressed narratives; using a fairly descriptive approach in order to minimise ‘overlaying’ their own interpretations on participants’ words. This approach was also consistent with the primary author’s belief that whether they have a mental health problem or not, young people are relatively proficient judges in identifying their own needs, and that they should be empowered and trusted in their experiences.
